# Effect of a Gender-Synchronized Family Planning Intervention on Inequitable Gender Norms in a Cluster Randomized Control Trial Among Husbands of Married Adolescent Girls in Dosso, Niger

**DOI:** 10.1177/10778012251366225

**Published:** 2025-08-11

**Authors:** Sabrina C. Boyce, Alexandra M. Minnis, Julianna Deardorff, Sandra I. McCoy, Sneha Challa, Nicole E. Johns, Sani Aliou, Mohamad I. Brooks, Abdoul-Moumouni Nouhou, Holly Baker, Jay G. Silverman

**Affiliations:** 1 Community Health Sciences Division, School of Public Health, 1438 University of California, Berkeley, Berkeley, CA, USA; 2 Women's Global Health Imperative, 6856 RTI International, San Francisco, CA, USA; 3 Division of Epidemiology, School of Public Health, 1438 University of California, Berkeley, Berkeley, CA, USA; 4 School of Nursing, 8785 University of California, San Francisco, San Francisco, CA, USA; 5 Center on Gender Equity and Health, School of Medicine, 8784 University of California, San Diego, La Jolla, CA, USA; 6 Niger Country Office, 14548 Pathfinder International, Niamey, Niger; 7 Headquarters, 14548 Pathfinder International, Watertown, MA, USA; 8 Niger Country Office, GRADE Africa, Niamey, Niger

**Keywords:** gender norms, social norms, cluster randomized control trial, Sub-Saharan Africa, couple-based family planning intervention

## Abstract

Gender inequitable norms have severe consequences for safety and health globally. Using data from a four-arm cluster randomized control trial of the Reaching Married Adolescents in Niger (RMA) intervention (2016–2019), this study assesses effects on gender norms among husbands of married adolescent girls (*n* = 1,055). Using an adjusted hierarchical difference-in-differences model, we found assignment to the RMA small groups intervention to be associated with a 0.62 decrease in inequitable gender norms (95% CI: −1.05, −0.18). As a cost-effective, scalable, and transferable intervention, this small group intervention could be valuable for reducing the negative impact of inequitable gender norms in similar settings.

## Introduction

Gender inequity is pervasive globally and has severe consequences for health and well-being (Heise, 2019). The relationship between gender inequity and related poor health may be especially clear in Niger, which ranks 154th of 162 countries on the United Nations Gender Inequality Index, based on reproductive health, empowerment, and economic activity (UNDP, 2020). With 76% of Nigerien girls marrying by the age of 18 and an adolescent birth rate of 177 births per 1,000 adolescents aged 15–19, early and frequent childbearing has contributed to an overall birth rate of 6.7 births per woman and maternal mortality ratio of 509 (per 100,000 live births) (The World Bank, 2020; UNICEF, 2018; UNPD, 2019; WHO, 2019). Early marriage and childbearing create family power structures in which married adolescent girls have minimal decision-making power relative to their older husbands and mothers-in-law and relatedly, limited ability to control their reproductive and sexual health, including the decision to use modern contraceptive methods (Fleming et al., 2020; Girls Not Brides, 2015; Tomar et al., 2021; UNICEF, 2014). Gender role expectations in this context confine married adolescent girls to the household, to care for the home and bear children early and often, and away from educational and leadership opportunities (Perlman et al., 2018). In Niger, as well as globally, shifts toward gender equity are viewed as crucial for societal well-being (Heise, 2019).

Gender inequity is embodied in the social norms of a society, which are expressed across all levels of Bronfenbrenner's socio-ecological framework (individual, social, institutional, and macro) (Bronfenbrenner, 2005; Cislaghi & Heise, 2019). Understood through social norms theory, social norms are the unspoken rules in a society about what behavior is acceptable and unacceptable (Mackie et al., 2015). In highly patriarchal contexts, gender norms, a subset of social norms that define gender-based social roles and behavior, often condone dominant forms of masculinity, female submissiveness, and male entitlement to control of women's bodies, even when these norms do not yield the best outcomes for the people involved (Bussey & Bandura, 1999; Cislaghi & Heise, 2017; Keleher & Franklin, 2008). The centrality of social norms, particularly gender norms, in shaping gender inequity and related practices is emphasized in multiple conceptual frameworks that inform behavioral interventions (Cislaghi & Heise, 2019; Heise, 1998; Pulerwitz et al., 2019). Gender norms and gender-based power simultaneously shape and are shaped by the institutions, resources, and social and individual systems of a society (Cislaghi & Heise, 2019).

Evidence suggests that public health interventions that change social norms, alongside other elements in the socioecology, can reduce the harmful health effects of gender inequity, but major gaps exist in understanding what intervention approaches and programs work to change social norms (Cislaghi & Heise, 2019; Dworkin et al., 2013; Haylock et al., 2016; Heise, 2019). There is growing interest in approaches that target social norms to change behaviour and reduce the health consequences associated with gender inequity (e.g., gender-based violence, child marriage, female genital cutting), especially in low- and middle-income countries (Cislaghi & Heise, 2019). For example, social norm interventions targeting both men and women, like Gender Equity Movement in Schools in India, Stepping Stones in South Africa, and Tostan in Senegal, have been shown to create behaviour changes that reduce intimate partner violence (IPV), herpes simplex 2 (HSV-2) infection, and female genital cutting, among other important health outcomes (Achyut et al., 2015; Jewkes et al., 2008; Rowley, 2020). Increasing interest in the involvement of men, often the decision-makers, gatekeepers, and perpetrators of violence against women, in gender norm-focused interventions is motivated by the potential of their involvement to disrupt and transform traditional power imbalances between men and women (Dworkin et al., 2013; Gupta, 2000). Synthesizing many of these learnings, the Social Norms Learning Collaborative (IRH, 2017) has proposed 10 key attributes for social norm-shifting interventions, including creating a safe space for critical community reflection, addressing power imbalances and inequity, and emphasizing the creation of a new positive norm. Little rigorous evidence exists, however, on social norms-focused interventions that have actually changed the targeted social norms, even when individual behaviour or the primary health outcomes are observed to improve.

Measurement of social norms is difficult and contributes to why program impact on social norms is rarely assessed (Cislaghi & Heise, 2016; Mackie et al., 2015). Cialdini et al.'s pivotal work around social norms has led to the classification of social norms as either *descriptive* (i.e., beliefs about what others do), *injunctive* (i.e., beliefs about how others would respond to one's behaviour), or *second-order beliefs* (i.e., beliefs about what others approve and disapprove of), all of which require careful consideration for accurate measurement (Chung & Rimal, 2016; Cialdini et al., 1991; Cialdini & Melanie, 1998; Mackie et al., 2015). In the rare cases in which effects on social norms are considered, aggregates of individual attitudes are often used as a proxy measure of social norms, which fails to capture the core element of social norms: perceived social expectations around behaviour (Cislaghi & Heise, 2016). The evaluation of SASA!, a community mobilization intervention to prevent violence and reduce HIV-risk behaviors, first implemented in Uganda, is justifiably acclaimed as one of the few cluster randomized control trials (cRCT) to assess and observe program effects on both social norms and behavioral outcomes, yet measurement of community-level social norms relied on aggregates of individual attitudes (Abramsky et al., 2014). The quasi-experimental evaluation of Voices of Change in Nigeria, a program that targeted young women and men to strengthen enabling environments for gender equality, assessed program effects on social norms using measures that include social acceptability of behavior, but effects were not detected (Denny et al., 2017). Similarly, the Saleema Initiative in Sudan, which aimed to promote community-level abandonment of female genital cutting, and Communities Care Programme in Somalia, which aimed to change social norms to end violence against women and girls in conflict-affected communities, showed quasi-experimental evidence of changing gender norms, with robust measurement of norms rather than aggregated attitudes, yet effects on the intended behaviors (female genital cutting and IPV, respectively) were not detected (Evans et al., 2019; Glass et al., 2019). As momentum to use social norm approaches in LMICs grows (Cislaghi & Heise, 2019), it is critical for researchers and practitioners to understand whether social norms-focused interventions actually change their intended social norms, and if so, whether behaviors informed by these norms subsequently change as a result. To advance this burgeoning field in public health research, such evidence is critical to supporting the development of programmatic conceptual frameworks and of social norm-shifting interventions.

The Reaching Married Adolescents in Niger (RMA) intervention, implemented starting in April 2017, aimed to address social norms and other barriers to increasing modern contraceptive use and reducing IPV among married adolescent girls and their husbands in the Dosso region of rural Niger. These primary behavioral outcomes were measured after receipt of 1 year of program implementation, and evidence of positive effects on both outcomes has been reported elsewhere (Silverman et al., 2022). Building on this evidence of behavior change as a result of the RMA intervention, the current study assesses intervention effects on gender norms among husbands, the primary decision-makers around contraceptive use, and perpetrators of IPV in cases when it occurs. Findings will help to close the gap in evidence on what works to change both gender norms and behavior in Niger and similar contexts.

## Methods

### Intervention

The RMA intervention is a gender-synchronized (i.e., concurrent programming for men and women), community-based program, developed and implemented by Pathfinder International among married adolescent girls (ages 13–19) and their husbands in the Dosso region of Niger to increase healthy birth spacing via the use of modern contraceptive methods. Program approaches, which were randomly assigned, were household visits or small group discussions; community-level dialogues were held concurrently in all intervention arms. Household visits were conducted by gender-matched, trained community health workers (CHWs) via 12 monthly visits to individual households for both married adolescent girls and their husbands. Household visits, provided to wives and their husbands separately, provided information and counseling on healthy timing and spacing of pregnancies and on access to and use of modern contraceptive methods. Small group discussions, also held separately by gender, were conducted by gender-matched CHWs trained to facilitate small groups and were held twice monthly for wives and once monthly for their husbands. Educational content delivered in these groups included general health and life skills, reproduction anatomy and health, use of modern contraceptive methods to accomplish healthy timing and spacing of pregnancies, gender norms that impede contraceptive use and female autonomy, couples’ communication regarding fertility decisions, and gender-based violence. Community dialogues were convened at the village-level each month by two trained facilitators to engage community gatekeepers and key influencers (e.g., traditional and community leaders, parents and in-laws) in creating an environment supportive of healthy timing and spacing of pregnancies and contraceptive use among married adolescent girls and their husbands (more details on the intervention, sample, and trial design are available elsewhere) (Challa et al., 2019). The intervention was implemented for 12 months.

### Research Study Design

We conducted a 4-arm factorial cRCT to evaluate the impact of receiving 1 year of the RMA intervention in 48 villages in the Loga, Doutchi, and Dosso districts in the Dosso region of Niger. A two-staged, stratified randomization strategy was utilized to accommodate the districts approved by the Niger Ministry of Health for inclusion and the limited feasibility of implementing more than one intervention model within a district. In the first randomization step, each of the three districts was randomly assigned a treatment condition (1:1:1). Arm 1 received household visits (Loga), Arm 2 received small group discussions (Doutchi), and Arm 3 received both (Dosso). All intervention arms also received community dialogues.

In the second randomization step, randomly selected villages within each district were assigned to either the treatment assigned to that district or the control group. Within each district, 16 villages were randomly selected for inclusion from villages meeting the inclusion criteria: (a) having at least 1,000 permanent inhabitants; (b) primarily Hausa or Zarma-speaking (the two major languages of Niger); and (c) not receiving any intervention specific to contraceptive use or gender equity in the past 2 years. Within each district, four of the 16 randomly selected villages were randomly assigned to the control group and the remaining 12 to the intervention condition assigned to that district, such that 12 villages were assigned to each study arm (1:1:1:1). Arm 4, therefore, consisted of the four control villages in each district (12 villages total). Random allocation was done by US-based study staff, with limited familiarity with the region at the time, by assigning numbers sequentially to an alphabetical list of the 16 villages and using a computer-generated random selection of four of those numbers for assignment to the control arm. The chief in each village provided a complete list of all households in which a married adolescent aged 13–19 years lived, and from this list, 25 wife-husband dyads were randomly selected for recruitment to the study, using a similar computer-generated random number selection process by US-based study staff. Local, trained research assistants visited the selected households to confirm eligibility (being Hausa or Zarma speaking; not planning to move away from the village in the next 18 months; not planning to travel away from the village for more than 3 months during that period; not sterilized; and providing informed consent) and random replacement was used if a household was not eligible or available.

The number of villages and wife-husband dyads selected for inclusion in the study was based on a power calculation for a minimum effect size on the main outcome, modern contraceptive use, of 2.0 or greater odds associated with the intervention. This calculation accounted for four arms with 12 clusters of equal size nested within each arm, time-by-treatment effects, an intra-class correlation kappa value of 0.05, attrition of 10%, and 80% power to detect an association of this power (Challa et al., 2019).

Data utilized in this analysis were collected at baseline (T1; May–June 2016) and 24-month follow-up (T2; April–June 2018). Full implementation of the intervention began 12 months after baseline data were collected, and T2 was conducted after 12 months of exposure to the intervention. At both time points, local research assistants who spoke Hausa or Zarma (depending on the village's language) conducted data collection using questionnaires programmed on tablet computers. Gender-matched research assistants interviewed married adolescent girls and husbands separately in a location deemed audibly private. The survey took 45–60 min to complete.

This trial was pre-registered on clinicaltrials.gov (NCT03226730) on July 24, 2017, with social norms regarding gender inequity defined as a secondary outcome. Ethics review boards of the University of California, San Diego School of Medicine (Project 160407S) and the Niger Ministry of Health (Project 011/2016/CCNE) approved all study protocols.

### Sample

There were 48 villages in the sample, with an average of 21 wife–husband dyads per village (range: 18–27 dyads). Only data provided by husbands were included in this analysis, as only participants who reported a male gender identity were asked the evaluated items on social norms regarding gender inequity. Data from female-identifying participants regarding RMA effects have been previously reported (Silverman et al., 2022).

### Measures

Perceived social norms regarding gender inequity (henceforward, SN-GEMS) were measured using a 5-item scale of second-order beliefs, a type of social norm, among husbands (Cronbach's alpha at T1 and T2: 0.62). The social norms measure was created for this study, adapted from the Gender Equitable Men Scale (GEMS), which has been used in more than 20 countries to assess male attitudes regarding gender equity (Singh et al., 2013). We adapted this scale by adding a stem to each dichotomous item that read, “People in my community think that…,” in order to measure second-order beliefs. The items then proceeded as, (a) “…a woman's most important role is to take care of the home and cook for the family”; (b) “…a man should have the final word about decisions in the home”; (c) “…there are times when a woman deserves to be beaten”; (d) “…a woman should never question her husband's decisions even if she disagrees with them”; (e) “…it is natural and right that men have more power than women in the family.” Response options included “agree,” “disagree,” “don’t know,” and “decline to answer.” Responses of “don’t know” and “decline to answer” were treated as missing for that item. Scores were calculated by adding up the number of affirmative responses a participant gave to the five items, with missing items assigned a zero value (range: 0–5); a higher score indicates higher perceived social acceptability of inequitable gender norms.

The study arm served as our main exposure variable (Arms 1, 2, 3, and the reference/control group). For sensitivity analyses to assess as-treated effects, we constructed a variable to categorize husbands who reported not receiving any sessions of the assigned RMA intervention in the past 12 months as “control” and husbands who reported receiving at least one session of the assigned RMA intervention in that intervention arm. Husbands in the control arm or an intervention arm reporting receipt of an intervention not assigned to that study arm were not considered treated, as geographic isolation of the intervention administration would have made that highly unlikely, and is more than likely a recall of exposure to a different intervention.

For sensitivity analyses to assess dose response, husbands were categorized as “no exposure” if they reported not receiving the intervention assigned to their district in the past 12 months, “low exposure” if they reported 1–3 sessions of the intervention assigned to their district, and “high exposure” if they reported 4+ sessions. For husbands in Arm 3 (combined intervention), exposure was defined by receipt of either intervention at these levels. Husband reports of exposure to an intervention not assigned to his district was not included as having received the intervention, as this is most likely a misreport of exposure to a nonrelated program.

Covariates were included in adjusted models to control for baseline socio-demographic differences between intervention and control arms. Factors considered were those that were identified to be associated with at least one intervention arm, via unadjusted multinomial logistic regressions for each factor (*p* < .05). Those that were selected were hypothesized, based on content expertise and theory, to also be related to the outcome. Covariates included husband's age, husband's and wife's education levels, husband's migration status, total household assets, and district (because intervention assignment was randomized at the district level). In calculating inverse probability of censoring weights (IPCW), used to minimize possible selection bias due to loss to follow-up, we also included covariates representing baseline differences between husbands retained and those lost to follow-up at T2. These covariates included husband's age (continuous), husband's age at marriage (continuous), husband's parity (continuous), husband's and wife's education level, and husband's migration status. *Husband's* and *wife's education levels* were categorically measured as ever receiving any modern/government school, any Quranic school (Muslim religious education), or no schooling (reference category). *Total household assets,* a measure of wealth, was measured as a count of how many of six listed items (e.g., mobile phone, bike, animal-drawn cart) any member of the household owned and included as a continuous variable (range: 0–6). *Husband's migration status* was dichotomously assessed as whether the husband spent more than 3 months away from the village in the past 12 months.

### Data Analysis

Descriptive Analyses. Descriptive analyses provided mean and standard deviations for continuous variables and prevalences for categorical variables. Differences in participation in the RMA intervention by arm were assessed using an unadjusted mixed effects logistic model clustering at the village level. Covariates and SN-GEMS scores were assessed for differences across study arms using an unadjusted mixed effects model regressing each study arm (reference group: control) on baseline characteristics.

Main Analyses. To account for the two-step randomization process, imbalances at baseline, and loss-to-follow-up, we assessed the intention-to-treat effect of the RMA intervention on husbands’ SN-GEMS score using a hierarchical difference-in-differences (DiD) linear regression. The DiD analysis approach was selected to account for known, meaningful differences at baseline across study arms. The primary model adjusted for covariates, accounted for village-level clustering in the study design and for multiple observations, and used IPCW to account for loss to follow-up. The model used robust standard errors to account for possible misspecification of the covariance structure.

Inverse probability of censoring weights were constructed by taking the inverse of the predicted probability of being retained in the study based on baseline demographic characteristics (Robins et al., 1995). Predicted probabilities were estimated using a logistic regression model that regressed study retention on covariates associated with loss to follow-up (covariates indicated for weights above). Missing weights were replaced with the median weight value. In the model, these weights rebalance the sample such that follow-up participants who had low predicted probabilities of being retained in the study are up-weighted, and those participants who had high predicted probabilities are down-weighted.

Sensitivity Analyses. The first set of sensitivity analyses was conducted to assess for effects in a model that preserves the randomization of the intervention and allows results to be interpreted as the population average effect across all villages (vs. within a given village, provided by the DiD results). For these analyses, we used a generalized estimating equation (GEE) to estimate the effect of the intervention arms on T2 SN-GEMS scores among husbands, using a Gaussian distribution, robust standard errors, an exchangeable correlation structure, controlling for baseline SN-GEMS scores, and clustering at the village level. The unadjusted model used only baseline SN-GEMS scores as a covariate and the adjusted model controlled for all covariates.

The second set of sensitivity analyses were conducted to assess for as-treated effects. If a husband received at least one session of the assigned intervention in the past 12 months, he was characterized as “treated” for that arm and if not, he was included in the control arm. We used the same DiD models to those used in the main analyses to estimate as-treated effects. Building upon this, we then assessed for a dose response to the RMA intervention, for husbands with no exposure, low exposure (1–3 sessions), or high exposure (4+ sessions). For these analyses, we used the same DiD model used in the main analyses, adjusted for covariates and IPCW, separately for each arm. The models were run with three subsamples: (a) all husbands in Arm 1 (household visits) and the control villages from that same district (Loga); (b) all husbands in Arm 2 (small groups) and control villages from the same district (Doutchi); (c) all husbands in Arm 3 (combo) and control villages from the same district (Dosso).

All analyses used STATA 15.1 (StataCorp, 2017). Significance was set at *p* < .05 for statistical testing, and estimates with 95% confidence intervals (CIs) are reported throughout.

## Results

**Figure 1.**
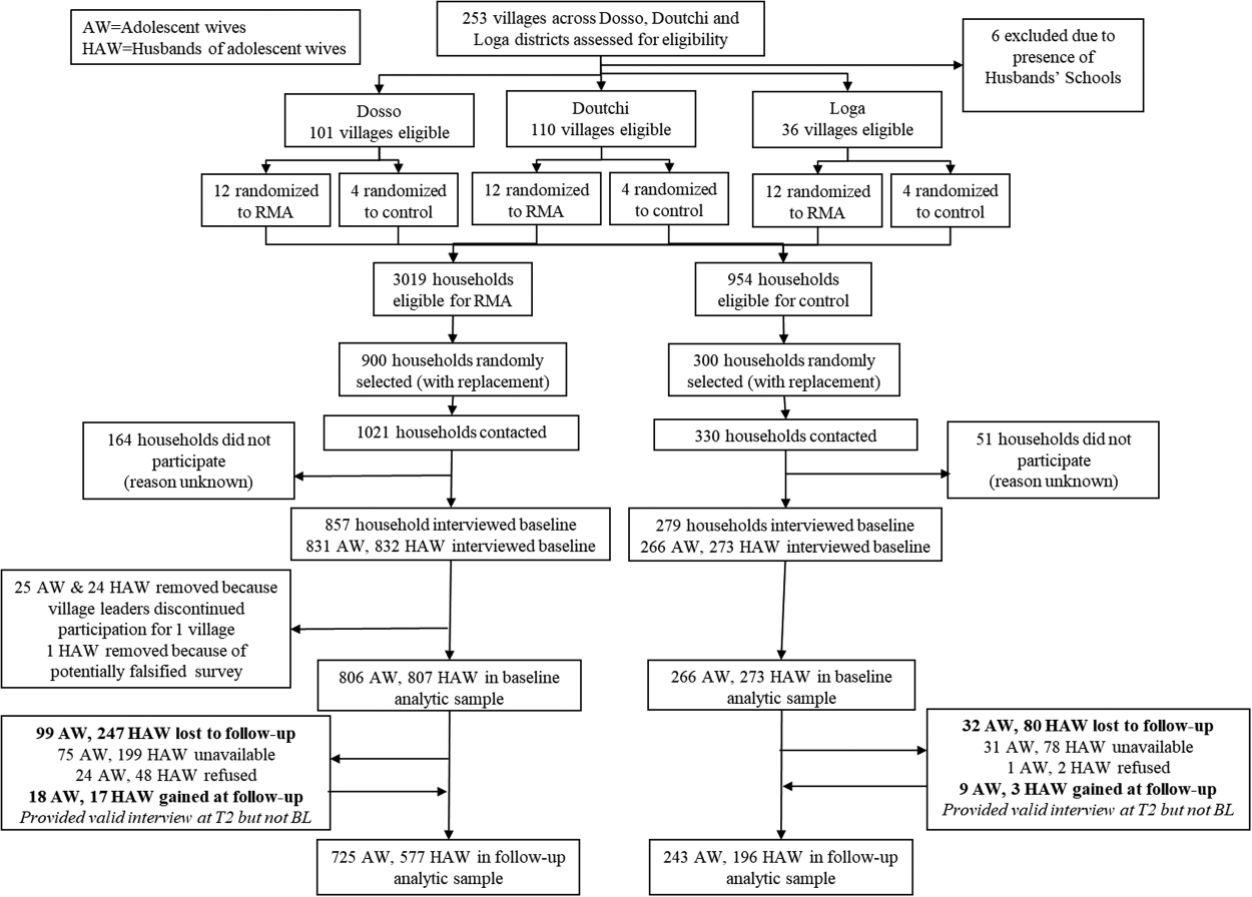
CONSORT Participant Flow for the Reaching Married Adolescents in Niger cluster randomized control trial (2016–2019).

At baseline, 1,080 of 1,351 eligible husbands completed surveys (79.9% male participation) and 773 participated in data collection at T2 (71.6% male retention) (Figure 1. CONSORT Participant Flow) (Silverman et al., 2022). On average, husbands were 25.6 (*SD* = 5.3) years old at baseline (Table 1). About one-third of husbands (29.4%) had never received any schooling. Husbands had 1.5 children on average and 67.2% migrated for more than 3 months in the past year. Households reported owning, on average, 2.1 (*SD* = 1.2) of the listed six assets.

**Table 1.** Husband Participation in the Reaching Married Adolescents in Niger Household Visits and Small Group Intervention Components at 24-Month Follow-Up (Dosso, Niger).

	RMA intervention arm			
Husband RMA program exposure	Arm 1: household visits (*n* = 205)	Arm 2: small groups (*n* = 194)	Arm 3: combination (*n* = 177)	*p*-value^a^
Participation in at least one RMA intervention component	146 (71.2%)	125 (64.4%)	134 (75.7%)	>.05
No household visits	59 (28.8%)	—	69 (39%)	
1–3 household visits	71 (34.6%)	—	62 (35%)	
4+ household visits	75 (36.6%)	—	46 (26%)	
No small groups	—	69 (35.6%)	60 (33.9%)	
1–3 small groups	—	66 (34%)	56 (31.6%)	
4+ small groups	—	59 (30.4%)	61 (34.5%)	

a *p*-values based on significance testing for differences in participation between intervention arms using an unadjusted mixed effects logistic model clustering at the village level with robust standard errors.

### Participation

Participation in 12 household visits and/or small groups (one per month for 1 year) represents full receipt of the possible RMA intervention. In Arm 1, 71.2% of husbands participated in at least one program element, 64.4% in Arm 2, and 75.7% in Arm 3 (Table 1). An unadjusted mixed effects logistic model clustering at the village level indicated a nonsignificant difference between participation in at least one program element across intervention arms, with the lowest participation among husbands in Arm 2 (*p* = .06). About one-third (29.7%) of the sample assigned to an intervention did not participate in any component of the evaluated intervention.

### Baseline Differences Across Study Arms

In comparing each intervention arm to the control arm, demographic characteristics varied (Table 2). Levels of education among husbands and wives were significantly different in all arms relative to controls. Arm 1 had lower Quranic education for men and women (*p* < .001) relative to control, Arm 2 had higher government and Quranic education (*p* < .001), and Arm 3 had lower Quranic education for women (*p* < .05) relative to controls. Total assets were lower in Arms 1 and 3 (*p* < .01 and *p* < .05, respectively) while Arm 2 had lower levels of husband migration (*p* < .001), relative to controls. Relative to controls, wives in Arm 2 were younger when married (13.8 years in Arm 2 vs. 14.3 years in control arm, *p* < 0.01) and at baseline (17.0 years in Arm 2 vs. 17.4 years among controls, *p* < .01) and husbands were older at baseline (26.5 years in Arm 2 vs. 25.5 years in control arm, *p* < .05; *results not shown*). In terms of the outcome, perceived inequitable gender norms were substantially lower at baseline in Arm 1 compared with the control arm (*p* < .001) (Table 2).

**Table 2.** Baseline Socio-Demographic Characteristics and Perceived Inequitable Gender Norms Among Married Adolescent Girls and Their Husbands Participating in the Reaching Married Adolescents Study in Dosso, Niger (*n* = 1,080 Husbands; *n* = 1,062 Wives).

	Overall (*n* = 1,062 wives; *n* = 1,080 husbands)		Arm 1 (household visits; *n* = 282)^a^		Arm 2 (small groups; *n* = 258)^a^		Arm 3 (combined; *n* = 267)^a^		Control arm (*n* = 273); reference group	
	Mean (*SD*)	*n* (%)	Mean (*SD*)	*n* (%)	Mean (*SD*)	*n* (%)	Mean (*SD*)	*n* (%)	Mean (*SD*)	*n* (%)
Husband age	25.6 (5.3)		25.7 (5.3)		26.5 (5.5)		24.9 (5.0)		25.5 (5.4)	
Wife age	17.3 (1.5)		17.4 (1.5)		17.0 (1.6)**		17.4 (1.5)		17.4 (1.5)	
Husband age at marriage	22.4 (5.0)		22.4 (4.7)		23.1 (5.4)		21.9 (4.8)		22.4 (5.0)	
Husband education										
Government school		522 (48.3)		149 (52.8)		136 (52.7)**		126 (45.8)		111 (40.7)
Quranic school		224 (20.7)		25 (8.9)**		80 (31.0)**		54 (19.6)		65 (23.8)
No school		326 (30.2)		108 (38.3)		40 (15.5)		83 (30.2)		95 (34.8)
Wife education										
Government school		382 (35.4)		89 (31.6)		109 (42.3)**		103 (37.5)		84 (30.8)
Quranic school		176 (16.3)		7 (2.5)**		84 (32.6)**		31 (11.3)		54 (19.8)
No school		514 (47.6)		185 (65.6)		63 (24.4)		137 (49.8)		134 (49.1)
Husband parity	1.5 (2.0)		1.6 (1.8)		1.4 (1.8)		1.4 (2.2)		1.43 (2.2)	
Total household assets (0–6)	2.1 (1.2)		1.9 (1.1)*		2.1 (1.2)		2.0 (1.2)*		2.2 (1.1)	
Husband migration (3+ months/year)		746 (69.1)		211 (74.8)		146 (54.9)		188 (70.4)		201 (73.6)
										
Husband Social Norms GEMS score (range: 0–5)										
T1	4.1 (1.1)		3.7 (1.4)**		4.3 (1.0)		4.2 (1.1)		4.2 (0.9)	
T2	4.0 (1.1)		4.0 (1.2)		3.7 (1.3)**		4.1 (1.0)		4.2 (0.9)	

Abbreviation: *SD* = standard deviation; GEMS = Gender Equitable Men Scale; T1 = baseline data collection; T2 = 24-month follow-up data collection.

a Significance testing conducted to detect baseline differences across study arms using an unadjusted multinomial logistic model regressing each study arm (reference group: control) on baseline characteristics using robust standard errors.

*p < .05, **p < .001

### Baseline Differences by Study Retention

Male retention in the study was associated with a variety of baseline factors. Husband study participants were more likely to be retained in the study if they were older (25.9 years among those retained vs. 25.0 years among those not retained at T2, *p* = .01), if they had more children with any wife (1.56 children vs. 1.26; *p* = .02), and if they did not migrate outside the village for more than 3 months in the previous 12 months (66.0% migrated vs. 77.6%; *p* < .001) [*results not shown*]. Husbands were also more likely to be retained in the study if their wives had received government or Quranic education (34.0% with education vs. 22.4%; *p* < .001) and if the age difference between husband and wife was larger (8.54 years difference vs. 7.72; *p* = .01). Study retention did not differ significantly by study arm (Arm 1: 71.0%, Arm 2: 72.9%, Arm 3: 64.4%, Arm 4: 70.3%, *p* = .15; data not shown). Factors associated with study retention were used in the creation of propensity weights.

### Time Trend

The mean SN-GEMS score at T1 was 4.1 [*n* = 1,055; range: 0–5; standard deviation (*SD*): 1.1] and T2 was 4.0 (*n* = 768; range: 0–5; *SD*: 1.1), indicating high perceived social acceptability of *inequitable* gender norms. Scores did not significantly differ between T1 and T2 in the entire sample nor among controls alone (mean score among controls at T1: 4.2 vs. at T2: 4.2); no overall temporal trend was detected (unadjusted *β*: 0.01, 95% confidence intervals (CI): −0.25, 0.28, *p* = .92) (Tables 2 and 3).

**Table 3.** Difference-in-Differences Effects of the Reaching Married Adolescents Intervention on Perceived Inequitable Gender Norms Among Husbands in Dosso, Niger (2016–2019).^a^

	Husbands (*n* = 1,090)					
	Unadjusted model with IPCW			Adjusted model with IPCW		
	*β*	95% CI	*p*-value	*β*	95% CI	*p*-value
Treatment Arm × Time Interaction						
Arm 1 (household visits)*T2	0.34	−0.13, 0.81	.16	0.32	−0.16, 0.80	.19
Arm 2 (small groups)*T2	−0.59	−1.02, −0.17	.01	−0.62	−1.05, −0.18	.01
Arm 3 (combined)*T2	−0.03	−0.38, 0.32	.85	−0.08	−0.42, 0.27	.67
Treatment arm						
Arm 1 (household visits)	−0.54	−0.96, −0.11	.02	−0.74	−1.13, −0.35	<.001
Arm 2 (small groups)	0.07	−0.24, 0.39	.66	0.32	−0.09, 0.72	.12
Arm 3 (combined)	−0.04	−0.38, 0.29	.81	−0.04	−0.38, 0.30	.83
Time (T1 to T2)	0.01	−0.25, 0.28	.92	0.03	−0.23, 0.30	.80
District (ref: Dosso)						
Doutchi	–	–	–	−0.26	−0.57, 0.06	.11
Loga	–	–	–	0.22	−0.005, 0.43	.05
Household assets	–	–	–	0.01	−0.05, 0.06	.8
Husband education (ref: no schooling)						
Government	–	–	–	0.0	−0.12, 0.14	.88
Quranic	–	–	–	0.06	−0.08, 0.20	.38
Wife education (ref: no schooling)						
Government	–	–	–	0.08	−0.05, 0.21	.23
Quranic	–	–	–	0.05	−0.12, 0.21	.58
Husband migration	–	–	–	0.04	−0.08, 0.17	.50
Husband age				−0.01	−0.02, 0.00	.05
						
Village-level standard deviation	0.10	–	–	0.11	–	–
Husband-level standard deviation	0.55	–	–	0.54	–	–
Residual intraclass correlation	0.57	–	–	0.55	–	–
Log pseudo-likelihood	−4,395.51	–	–	−4,291.54	–	–

Abbreviations: IPCW = inverse probability of censoring weights; CI = confidence interval; T1 = baseline data collection; T2 = 24-month follow-up data collection.

a Unadjusted and adjusted difference in differences effects were estimated using random intercept linear regression models with an interaction term for time*study arm and inverse probability of censoring weighting.

### Multilevel Modeling

In both unadjusted and adjusted difference-in-differences models, we found assignment to the small group RMA intervention (Arm 2) to be associated with a significant decrease in perceived social acceptability of inequitable gender norms at follow-up (Table 3). Assignment to RMA small groups among husbands within a given village was associated with a 0.62 lower score on the SN-GEMS (95% CI: −1.05, −0.18) relative to controls, after adjustment for baseline differences. The latter represents 0.56 of a standard deviation change in score. The household visit intervention (Arm 1) was associated with an increase in inequitable gender norms, but this estimate was not significant and imprecise with wide confidence intervals (Table 3). Assignment to the RMA household visits among husbands within a given village was associated with a 0.32 higher score on the SN-GEMS (95% CI: −0.16, 0.80) relative to controls, after adjustment for baseline differences. No significant effects on gender norms were detected for the RMA combination intervention (*β*: −0.08, 95% CI: −0.42, 0.27).

### Sensitivity Analyses: GEE Models Preserving Randomization

We conducted sensitivity analyses to understand RMA effects on SN-GEMS scores at follow-up using GEE models, which preserve the randomization of districts to intervention arm and villages to the assigned intervention or control and allow for interpretation of results across all villages (rather than within a village, as in the above random effects models). In unadjusted and adjusted GEE models, we found similar, though slightly attenuated results (Table 4). Random assignment to the small group (Arm 2) was associated with a 0.33 lower SN-GEMS score (95% CI: −0.52, −0.14) at follow-up relative to controls across all villages, after adjusting for baseline differences. No significant difference in SN-GEMS scores at follow-up were observed for household visits (Arm 1) or the combination (Arm 3) interventions relative to controls across all villages.

**Table 4.** Sensitivity Analysis: Estimated Effects of the Reaching Married Adolescents Intervention on Perceived Inequitable Gender Norms Using a Generalized Estimating Equation Among Husbands in Dosso, Niger (2016–2019).^a^

	Husbands (*n* = 1,090)					
	Unadjusted GEE			Adjusted GEE		
	*β*	95% CI	*p*-value	*β*	95% CI	*p*-value
Treatment arm						
Arm 1 (household visits)	−0.09	−0.4, 0.22	.56	−0.15	−0.81, 0.5	.65
Arm 2 (small groups)	−0.49	−0.77, −0.21	.00	−0.33	−0.52, −0.14	.00
Arm 3 (combined)	−0.05	−0.35, 0.24	.74	−0.15	−0.41, 0.1	.23
T1 Social Norms GEM Score	0.06	−0.02, 0.14	.15	0.05	−0.03, 0.14	.23
District (ref: Dosso)						
Doutchi	–	–	–	−0.31	−0.47, −0.16	.00
Loga	–	–	–	−0.01	−0.63, 0.62	.98
Household assets	–	–	–	0.02	−0.05, 0.08	.61
Husband education (ref: no schooling)						
Government	–	–	–	0.08	−0.1, 0.26	.39
Coranic	–	–	–	0.12	−0.1, 0.34	.27
Wife education (ref: no schooling)						
Government	–	–	–	0.14	−0.05, 0.32	.15
Coranic	–	–	–	0.21	−0.07, 0.48	.15
Husband migration	–	–	–	0.16	−0.03, 0.34	.10
Husband age				−0.01	−0.02, 0.01	.40

Abbreviations: CI = confidence interval; GEE = generalized estimating equation; T1 = baseline data collection; T2 = 24-month follow-up data collection.

a Unadjusted and adjusted effects were estimated using a generalized estimating equation controlling for baseline SN-GEMs scores clustering on village, preserving the original randomization of treatment.

### Sensitivity Analyses: As-Treated

We conducted sensitivity analyses to understand RMA effects on SN-GEMS scores when we categorized husbands based on their actual participation in the RMA intervention assigned to their district. Those who reported participating in at least one of the intervention components assigned to their district we considered treated and those who reported no participation as a control. In adjusted DiD analyses based on exposure to the assigned intervention, we found similar results to those found in the primary analyses: null effects on SN-GEMS for those husbands in Arm 1 exposed to household visits (*β*: 0.32, 95% CI: −0.16, 0.80), a significant decrease in SN-GEMS scores among husbands in Arm 2 exposed to small groups (*β*: −0.62, 95% CI: −1.05, −0.18), and null effects among husbands in Arm 3 exposed to at least one of the intervention components (*β*: −0.08, 95% CI: −0.42, 0.27) (Table 5). As-treated analyses should be interpreted with some caution because RMA is a village-level intervention that likely spills over to all residence of a village due to the community-level intervention components, regardless of self-reported exposure.

**Table 5.** Sensitivity Analyses: As-Treated Difference-in-Differences Effects of the Reaching Married Adolescents Intervention on Perceived Inequitable Gender Norms Among Husbands in Dosso, Niger (2016–2019).^a^

	Husbands (*n* = 1,055)		
	Adjusted model with IPCW		
	*β*	95% CI	*p*-value
Treatment Arm × Time Interaction			
Arm 1 (household visits)*T2	0.32	−0.16, 0.80	.19
Arm 2 (small groups)*T2	−0.62	−1.05, −0.18	.01
Arm 3 (combined)*T2	−0.08	−0.42, 0.27	.66
Treatment arm			
Arm 1 (household visits)	−0.74	−1.13, −0.35	.00
Arm 2 (small groups)	0.32	−0.09, 0.72	.12
Arm 3 (combined)	−0.04	−0.38, 0.30	.83
Time (T1 to T2)	0.03	−0.23, 0.30	.80
District (ref: Dosso)			
Doutchi	−0.26	−0.57, 0.06	.11
Loga	0.22	0, 0.43	.05
Household assets	0.01	−0.05, 0.06	.80
Husband education (ref: no schooling)			
Government	0.01	−0.12, 0.14	.88
Quranic	0.06	−0.08, 0.20	.38
Wife education (ref: no schooling)			
Government	0.08	−0.05, 0.21	.23
Quranic	0.05	−0.12, 0.21	.58
Husband migration	0.04	−0.08, 0.17	.50
Husband age	−0.01	−0.02, 0.00	.05
log pseudo-likelihood	−4291.54	–	–

Abbreviations: IPCW = inverse probability of censoring weights; CI = confidence interval; T1 = baseline data collection; T2 = 24-month follow-up data collection.

a Adjusted difference in differences effects were estimated using random intercept linear regression models with an interaction term for time*study arm and inverse probability of censoring weighting.

**Table 6.** Sensitivity Analyses: Dose-Response Effects of the Reaching Married Adolescents Intervention on Perceived Inequitable Gender Norms Among Husbands in Dosso, Niger (2016–2019).^a^

	Arm 1 (household visit; *n* = 292)			Arm 2 (small group; *n* = 283)			Arm 3 (combined; *n* = 292)		
	Adjusted			Adjusted			Adjusted		
	*β*	95% CI	*p*-value	*β*	95% CI	*p*-value	*β*	95% CI	*p*-value
Model 1: Difference in Differences Model for Treatment Dose × Time Interaction^a^									
No exposure * T2	Ref			Ref			Ref		
Low exposure * T2	0.46	−0.09, 1.02	.10	−0.42	−0.88, 0.04	.08	−0.11	−0.48, 0.26	.56
High exposure *T2	0.15	−0.42, 0.72	.61	−0.92	−1.54, −0.3	.003	−0.20	−0.72, 0.33	.46
Model 2: GEE model for treatment dose^b^									
No exposure	Ref			Ref			Ref		
Low exposure	−0.02	−0.30, 0.25	.86	−0.22	−0.56, 0.12	.20	−0.16	−0.42, 0.11	.24
High exposure	−0.28	−0.65, 0.10	.15	−0.81	−1.38, −0.23	.01	−0.25	−0.45, −0.04	.02

Abbreviations: CI = confidence interval; GEE = generalized estimating equation; T2 = 24-month follow-up data collection.

a Model 1 was run for each intervention arm and utilized the adjusted difference-in-differences model used in main analyses. This model is a random intercept linear regression model with an interaction term for time*study arm and inverse probability of censoring weighting.

b Model 2 was also run for each intervention arm and utilized the generalized estimating equation that preserves the intervention randomization. The model was run with a Gaussian distribution controlling for baseline SNGEM scores clustering on village, preserving the original randomization of treatment.

### Sensitivity Analyses: Dose Response

Our final sensitivity analyses build upon results from the as-treated DiD results to assess for a dose-response of exposure to the RMA interventions on SN-GEMS. We assessed for dose-response using both DiD models and GEE models, the latter because analyses were conducted separately for each arm with only control villages from the same district, and randomization was more successful in terms of baseline differences. In unadjusted and adjusted DiD models, we found a dose-response relationship with the small group intervention (Arm 2) (Table 6). Compared to husbands in control villages in the Doutchi district, husbands who received low exposure to small groups (1–3 sessions) over the previous 12 months had −0.42 lower SN-GEMS scores (95% CI: −0.88, 0.04) and husbands who received high exposure (4+ group sessions) had −0.92 lower SN-GEMS scores (95% CI: −1.54, −0.30) within a given village. The latter represents 0.83 of a standard deviation change in score. No dose response was observed for Arms 1 and 3.

In unadjusted and adjusted GEE models, we found a dose-response relationship with the small group intervention (Arm 2) and the combined intervention (Arm 3). Compared to husbands in control villages in the Doutchi district, husbands who received low exposure to small groups (1–3 sessions) had −0.22 lower SN-GEMS scores (95% CI: −0.56, 0.12) and husbands who received high exposure (4+ group sessions) had −0.81 lower SN-GEMS scores (95% CI: −1.38, −0.23) across all villages. Compared to husbands in control villages in the Loga district, husbands who received low exposure (1–3 sessions) to either small groups or household visits had −0.16 lower SN-GEMS scores (95% CI: −0.42, 0.11) and husbands who received high exposure (4+ sessions) had −0.25 lower SN-GEMS scores (95% CI: −0.45, −0.04) across all villages. No dose response for Arm 1 was observed in GEE models.

## Discussion

Results from this cRCT suggest that the small group RMA intervention decreased social norms supporting gender inequitable behavior among husbands of married adolescent girls in the Dosso region of Niger. Across main analyses and all sensitivity analyses, the effect of the small group intervention on social norms was robust. Only a limited number of cRCTs and quasi-experimental evaluations of social norm-focused interventions in LMIC contexts have shown direct programmatic effects on social norms (Abramsky et al., 2014; Evans et al., 2019; Rowley, 2020). Even fewer studies have demonstrated direct effects on both social norms and behavioral outcomes (Abramsky et al., 2014). The present study builds on previous evidence (using the same evaluation data) indicating that the small group RMA intervention reduced IPV among married adolescent girls (Silverman et al., 2022). The present study is the first to demonstrate that the RMA small group intervention not only resulted in this important behavioral outcome, but it also reduced gender inequitable social norms.

Moreover, results from sensitivity analyses indicate that there may be a critical dose-response relationship between the small group intervention and changes in perceived social norms among husbands, a criterion for establishing causality. While a low dose of the small group intervention showed a trend toward improved social norms, a high dose (4+ sessions) provided over double the effect. In dose-response GEE models that preserved randomization only, a dose-response relationship was also observed for the combination arm, such that improvements in social norms were significantly associated with a high dosage of either intervention. Of important note to practitioners, these results indicate that the greatest benefits of the small group and combination interventions occur with exposure to at least four sessions in 12 months.

Despite the entrenched, deep-seated nature of social norms, the RMA small group intervention approach was successful in creating significant positive shifts in these norms, with greater exposure leading to greater changes in norms. Given the high mean SN-GEMS scores at baseline and follow-up (4.1 and 4.0, respectively, indicating high perceived social acceptance of gender inequitable social norms), a decrease in score of the magnitude observed for exposure to four or more sessions of the small group (approximately one standard deviation below the mean) suggests a strong departure from the status quo and a practically meaningful difference in perceived social norms. The magnitude of the effects on social norms found in this study is larger than those found in previous research. For example, the Saleema Initiative, which aimed to shift norms related to FGM, found with their 4-item scale on social norms a 0.33 reduction in scores as a result of the intervention, while in the present study high exposure to the small group intervention was associated with a 0.81 reduction on a 5-item scale (Evans et al., 2019).

The small group model, facilitated by local CHWs, aimed to provide education and a space for men to gather, discuss, and possibly challenge one another's thinking around gender roles. The resulting shifts in norms observed in this study provide empirical evidence to support current theoretical understanding of how social norms change; social norms are socially constructed concepts of acceptable behavior within a community and are most amenable to change in social contexts. Other interventions that have been shown to change social norms or aggregated individual attitudes, like SASA!, also utilize male discussion groups to shift social norms, but typically also intensively engage the wider community through mobilizing efforts (Abramsky et al., 2014; Cislaghi & Heise, 2018). The RMA intervention offers a simple, cost-effective approach to social norms change that is feasible to implement in one of the most resource-limited settings among LMICs.

Social norms interventions have been shown to be effective when they provide information or correct misinformation about peer's behaviors or provide space for renegotiation of social norms within a social group (Miller & Prentice, 2016). It aligns, then, that receipt of RMA household visits, an intervention delivered to each individual of a married couple without information about peer behavior or opportunity for the exchange of ideas among peers, did not show evidence of changing perceived inequitable gender norms, yet, importantly, did result in the desired behavior change—contraceptive use. The RMA intervention, including household visits, was designed with the aim of increasing contraceptive use (i.e., social norms were not the primary outcome), and evidence indicates household visits were effective at doing so (Silverman et al., 2022). The nonsignificant effect on perceived inequitable gender norms associated with household visits, though, suggests that these visits provided little opportunity for husbands to see change within their peers around issues of gender equity. Similar null effects were observed in the combination arm for all analyses except GEE dose-response models, which found some evidence of a dose-response effect on social norms. As the combination arm offered two intervention components (household visits and small groups) with opposing effects on social norms, these effects likely counter-balanced one another, resulting in no effect on social norms in this arm in the majority of analyses. Yet, our previous research indicates positive behavior change for all three intervention arms, emphasizing the utility of both the household visit and small group intervention for contraceptive use and IPV, respectively, even in the absence of social norms change (Silverman et al., 2022).

This study is not without limitations. First, despite treatment randomization, baseline differences in treatment and control arms exist because the first level of randomization was linked to the district. This design decision was due to real-world constraints of randomization, which, in this case, came from the Ministry of Health's requirement that randomization be conducted at the district level. Baseline differences likely resulted from important social, economic, and cultural differences among district residence. To address this, control villages were randomly selected from all of the three intervention districts such that each district is represented by a third of the control villages. We also utilized a difference-in-difference analytical approach and controlled for district and other baseline differences, mostly related to randomization at the district level. Still, unmeasured confounding may obscure these results. For example, at baseline, Arm 1 had significantly lower levels of Quranic schooling relative to controls. Importantly, this arm also had significantly lower scores on the SN-GEMS at baseline relative to controls, suggesting lower acceptance of inequitable gender norms relative to other arms, which could be related to the lower levels of Quranic education in these villages, and may contribute to the imprecise estimation of effects on social norms for Arm 1. More scrutiny of these and other factors that may have contributed to unmeasured confounding is needed to confirm results on RMA household visits.

Second, there was a notable loss to follow-up among husband participants at T2, which could have led to selection bias. To minimize the impact of this source of selection bias, we utilized IPCW in our models to re-weight the data so that it more closely approximates the data had those participants not been lost. Third, the interpretation of random intercept models is the change in score between individuals receiving treatment and those receiving control *within a cluster*. In this study design, all residents in a village received either treatment or control, never a mix of the two. Therefore, the random effects model interpretations reflect a theoretical within village effect that was not possible in reality. We conducted sensitivity analyses using GEE that preserve the randomization of the interventions and allowed for interpretation *across all villages*. Results from these analyses upheld the main analysis's findings.

Third, measurement of social norms is novel and underdeveloped. This study used a novel scale for measuring gender inequitable social norms adapted from a well-validated attitudinal scale. It is likely that not all aspects of gender norms are captured in this scale, and possible that the measurement of norms is imprecise, obscuring some of the true effects of the intervention. Finally, these results only represent the effects of the RMA intervention on inequitable gender norms in a probability sample of husbands of married adolescent girls in the rural Dosso region of Niger. While these results are not representative of other regions in Sub-Saharan Africa, they do suggest that if a positive impact can be achieved in this setting, the small-group elements of the RMA intervention may be promising in other similarly resourced, culturally related settings. Future research in other settings is needed to assess if effects can be replicated.

## Conclusions

These results provide preliminary evidence that the RMA small group intervention approach is effective at reducing inequitable gender norms and behavior change, one of the very few interventions in LMIC demonstrating such effects. While further careful investigation of unmeasured confounding is needed, these results also suggest that the household visit and combination RMA intervention did not change perceptions of inequitable gender norms, despite increasing contraceptive use, possibly due to the lack of facilitated social exchange in household visits. Inequitable gender norms are understood to be an important, though difficult to change, upstream risk factor for a variety of adverse health outcomes, including IPV, reproductive coercion, child marriage, and maternal mortality. The potential ripple effect of an intervention that decreases inequitable gender norms among this high-priority population of husbands of married adolescent girls in Niger could provide long-term benefit across multiple related health outcomes. Moreover, as a cost-effective, simple, scalable, and transferable intervention with evidence of being able to change such gender norms, this CHW-based small group intervention approach could be extremely valuable to the field of public health for reducing the negative impact of inequitable gender norms on health and well-being in similar settings.
